# A novel rotation and scale-invariant deep learning framework leveraging conical transformers for precise differentiation between meningioma and solitary fibrous tumor

**DOI:** 10.1016/j.jpi.2025.100422

**Published:** 2025-02-04

**Authors:** Mohamed T. Azam, Hossam Magdy Balaha, Akshitkumar Mistry, Khadiga M. Ali, Bret C. Mobley, Nalin Leelatian, Sanjay Bhatia, Murat Gokden, Norman Lehman, Mohammed Ghazal, Ayman El-Baz, Dibson D. Gondim

**Affiliations:** a BioImaging Lab, Department of Bioengineering, University of Louisville, Louisville, KY, USA; b Department of Neurosurgery, University of Louisville School of Medicine, Louisville, KY, USA; c Department of Pathology, Faculty of Medicine, Mansoura University, Mansoura, Egypt; d Department of Pathology, Vanderbilt University Medical Center, Nashville, TN, USA; e Department of Neurosurgery, West Virginia University, Morgantown, WV, USA; f Department of Pathology, University of Arkansas for Medical Sciences, Little Rock, AR, USA; g Department of Pathology, Baylor Scott & White Hospital, Baylor College of Medicine, Temple, TX 76508, USA; h Electrical, Computer, and Biomedical Engineering Department, Abu Dhabi University, Abu Dhabi, United Arab Emirates; i Department of Pathology, University of Louisville School of Medicine, Louisville, KY, USA

**Keywords:** Computer aided diagnosis (CAD), Histopathology, Meningioma (MEN), Solitary fibrous tumors (SFT), Vision transformer (ViT)

## Abstract

Meningiomas, the most prevalent tumors of the central nervous system, can have overlapping histopathological features with solitary fibrous tumors (SFT), presenting a significant diagnostic challenge. Accurate differentiation between these two diagnoses is crucial for optimal medical management. Currently, immunohistochemistry and molecular techniques are the methods of choice for distinguishing between them; however, these techniques are expensive and not universally available. In this article, we propose a rotational and scale-invariant deep learning framework to enable accurate discrimination between these two tumor types. The proposed framework employs a novel architecture of conical transformers to capture both global and local imaging markers from whole-slide images, accommodating variations across different magnification scales. A weighted majority voting schema is utilized to combine individual scale decisions, ultimately producing a complementary and more accurate diagnostic outcome. A dataset comprising 92 patients (46 with meningioma and 46 with SFT) was used for evaluation. The experimental results demonstrate robust performance across different validation methods. In train-test evaluation, the model achieved 92.27% accuracy, 87.77% sensitivity, 97.55% specificity, and 92.46% F1-score. Performance further improved in 4-fold cross-validation, achieving 94.68% accuracy, 96.05% sensitivity, 93.11% specificity, and 95.07% F1-score. These findings highlight the potential of AI-based diagnostic approaches for precise differentiation between meningioma and SFT, paving the way for innovative diagnostic tools in pathology.

## Introduction

Meningioma (MEN) and solitary fibrous tumor (SFT) are two kinds of tumors that are differentiated by their histological characteristics, origins, and clinical significance.^1^ MEN is primarily a benign growth that arises from the meninges, the protective layers surrounding the brain and spinal cord. They represent one of the more frequent types of primary brain tumors in adults.^2^ Although most MENs carry a favorable prognosis, they may cause symptoms as they enlarge and put pressure on adjacent brain structures. Some of the histological patterns encountered in MENs share overlapping characteristics with other types of tumors, which often create difficulties in their diagnosis and classification.^3^ SFT are rare mesenchymal tumors that may originate in various anatomical locations, such as soft tissues and organs. SFTs can also involve central nervous system meninges, which are typical sites for MENs. In the central nervous system, SFT have high propensity for aggressive behavior in the form of recurrence and metastasis, which can occur decades after the diagnosis.^4^ The histopathological overlap between MENs and SFTs poses a significant challenge in distinguishing between them. The presence of specific molecular alterations in SFTs is a key feature used to resolve this differential diagnosis. SFTs are molecularly defined by a genomic inversion at the 12q13 locus, leading to the fusion of the NAB2 and STAT6 genes, which results in STAT6 nuclear expression. This can be evaluated by both molecular testing, which detects the specific alteration, and immunohistochemistry (IHC), which identifies the abnormal expression of the STAT6 protein within the nuclei of neoplastic cells.

IHC is an important technique in histopathology for characterizing protein or antigen expression in tissue samples by using antibodies to identify and visualize proteins within tissue sections.^5^ However, IHC faces significant challenges due to its complex infrastructure requirements, including specialized instruments, consumables, validation procedure, and highly trained personnel, leading to high operational costs. Maintaining a large menu of antibodies is costly, and smaller labs do not offer infrequently used antibodies, such as STAT6, resulting in samples being sent to reference labs, increasing costs and delays. This process extends diagnosis time, impacting patient management and treatment decisions. Additionally, most labs cannot perform IHC on frozen sections, critical for providing rapid diagnostic information during surgeries.^6^ Molecular testing shares similar infrastructure and operational complexities as IHC, typically takes longer, and is more expensive, further increasing costs for patients.^7^ Therefore, there is a high demand to find alternative methods to gain accurate and reliable diagnostic assessments.^1^

Some limitations could be addressed by utilizing image analysis and artificial intelligence (AI) as alternative diagnostic methods.^8^ By employing machine learning and deep learning algorithms, it is possible to detect and classify distinctive tissue characteristics and arrangements that can support the diagnostic process.^9^

In the field of radiology, there is a body of literature addressing the differential diagnosis of MENs and SFTs using AI.^10^, ^11^, ^12^, ^13^, ^14^ However, the final diagnostic distinction requires histopathological analysis. To our knowledge, there is no prior work creating an AI classifier to distinguish between these two diagnoses based on histopathology.

This research article explores the feasibility of weakly supervised transformer-based models for diagnosing MEN and SFT tumors on histopathology and opens potential improvements in terms of the accuracy of diagnosis, especially where ordinary approaches can be hindered. The proposed solution takes full advantage of the strengths of vision transformers in histopathology. The objective of this research is to design a novel architecture of conical transformers at multiple whole-slide image (WSI) magnification levels that capture the various global and local imaging markers across the optical scales. The outputs from these transformers are then combined using a weighted majority voting (WMV) scheme to attain a complementary and more reliable diagnosis. The contribution to this study can be described as follows:

• **Transformer-based approach**: A new approach is proposed through weakly supervised transformer classifiers applied in WSIs, giving a promising alternative to the conventional diagnostic approach.
• **Rotation and scale-invariant approach**: An architecture of conical transformers applied at multiple magnification scales is designed to encompass the hierarchical nature of WSIs.
• **Progress in AI-driven histopathology**: This technique could enhance the diagnostic process by applying AI-based approach to imaging, decreasing costs and avoiding delays caused by techniques such as IHC and molecular testing.

### Article organization

The rest of this article is organized as follows: Materials introduce the research data and resources. Methodology outlines the approach and techniques employed. Experimental results present the experiment's setup and findings. Overall discussion analyzes the key outcomes of the study. Limitations highlight the constraints of this work. Finally, conclusions and future directions summarize the article and propose avenues for future research.

## Materials

**Fig. 1:**
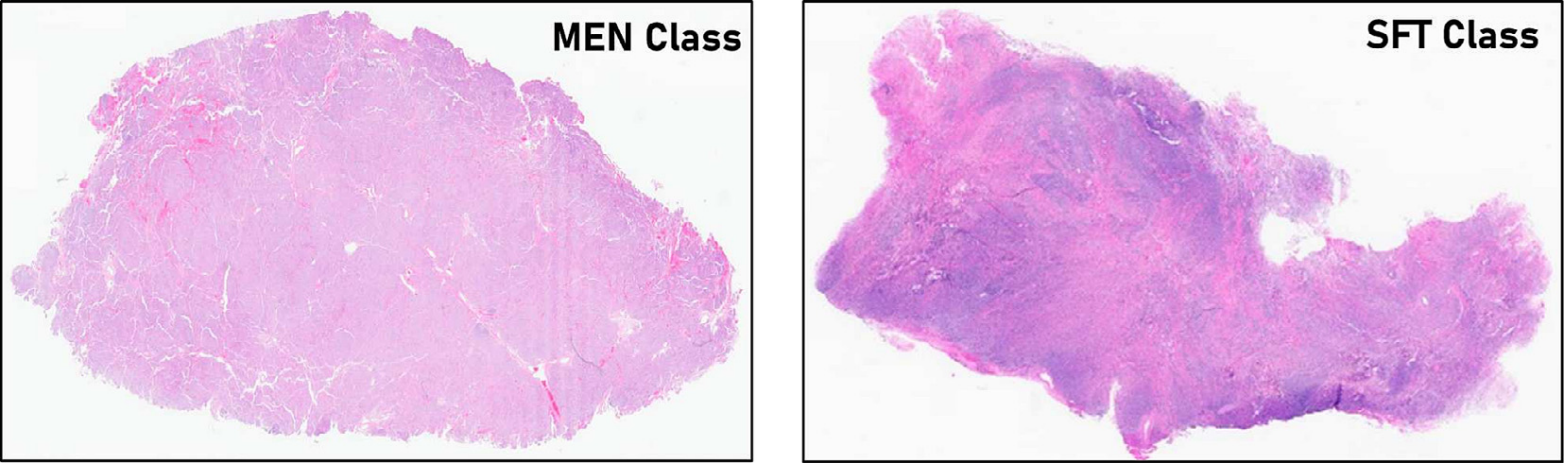
Presentation of histopathological samples showcasing (Left) meningioma (MEN) and (Right) solitary fibrous tumor (SFT) classes from the dataset.

The study utilized a dataset sourced from multiple institutions consisting of 46 WSIs of MEN cases and 46 WSIs of SFT cases. Expert pathologists provided the original diagnoses and conducted a meticulous review of all pathology reports and histology slides. SFT diagnoses were confirmed using STAT-6 IHC. A single representative hematoxylin and eosin (H&E) slide per case was selected, which was subsequently digitized into WSIs using the GT450 scanners with a 40× resolution and 0.25 μm/pixel. The resulting WSIs are multi-level SVS images equipped with pyramidal structures, enabling magnification capabilities of up to 400×. Fig. 1 presents visual representations of typical samples from both classes offering a representative glimpse into the dataset's contents.

## Methodology

**Fig. 2:**
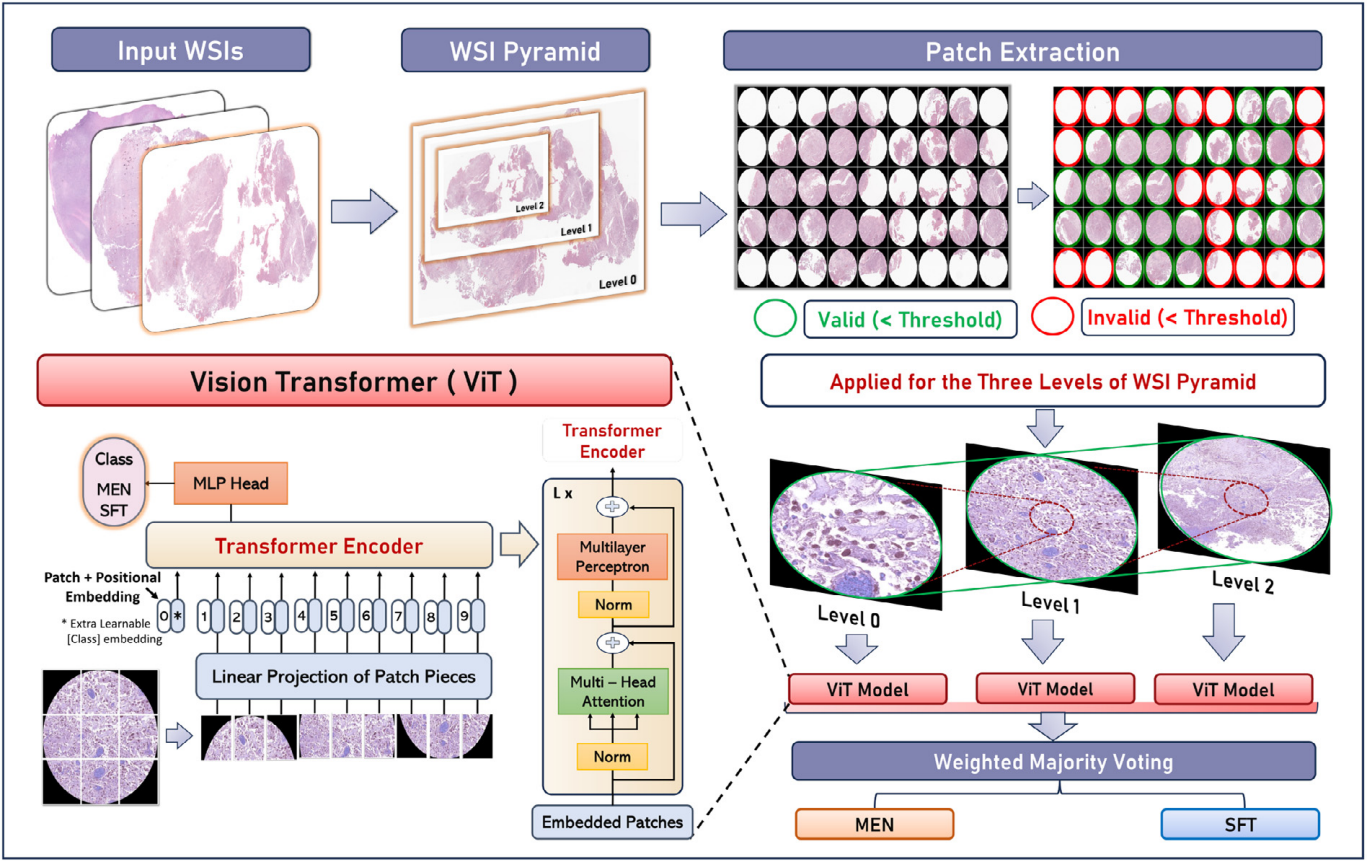
Illustration of the entire pipeline workflow, including input WSIs, patch-level extraction, and filtering. The process involves feeding data into distinct Vision Transformers (ViTs), followed by a majority weighting schema for final classification. Additionally, an overview of the ViT model architecture is provided.

The proposed framework begins with the extraction of patches from the three different levels in the WSI hierarchy. Those regions of patches that contain very little histological information, probably due to high background or blood products, are identified and excluded. The remaining high-quality patches from every WSI level are fed into a ViT model, namely the Large-32 variant to perform the classification process. To aggregate results from multiple ViT models, a WMV scheme has been utilized. Fig. 2 shows an in-depth visual representation of the entire system pipeline.

### WSI levels separation

**Fig. 3:**
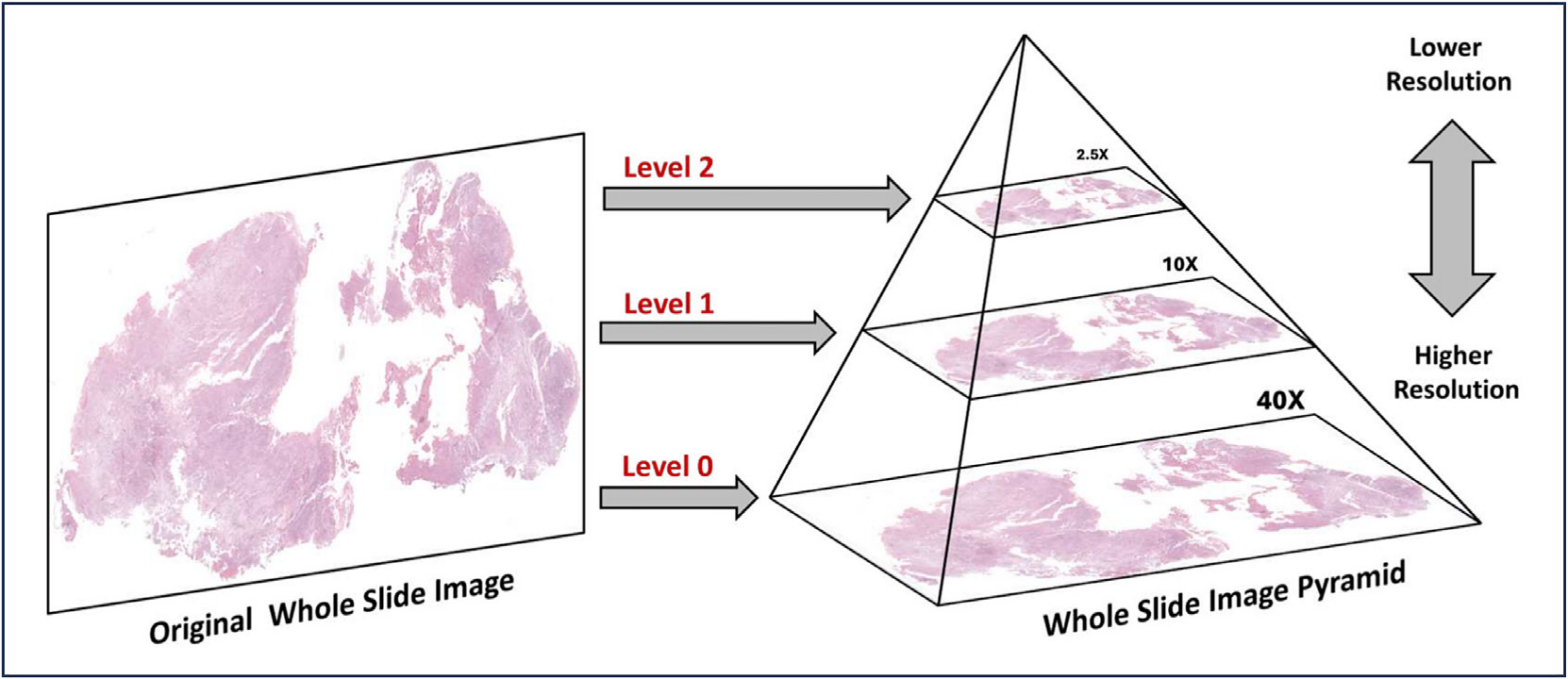
Illustration illustrating the hierarchical division of WSIs into discernible levels: Level 0 (40× magnification), Level 1 (10× magnification), and Level 2 (2.5× magnification).

The acquired WSIs were stored in the SVS format. Each SVS image underwent a deliberate process of decomposition into three discernible levels, designated as Level 0, Level 1, and Level 2, corresponding to magnifications of 40×, 10×, and 2.5×, respectively. This separation was necessary to facilitate multi-resolution analysis as each level was supposed to carry a different amount of detail. Level 0 has the highest resolution capability, detailing the fine structures of cells and pathological features. Level 1 has a medium resolution, offering a balanced view by capturing both localized details and broader tissue patterns and Level 2 is able to give a panoramic view, capturing larger-scale patterns and the overall tissue arrangement with its corresponding low resolution. This hierarchical presentation logically guided traversal of the different tiers of detail contained within the histopathological images, so that all relevant information could be easily accessed for further analysis. Fig. 3 shows a graphical representation of the WSI-level separation process.

### Patches extraction and preprocessing

**Fig. 4:**
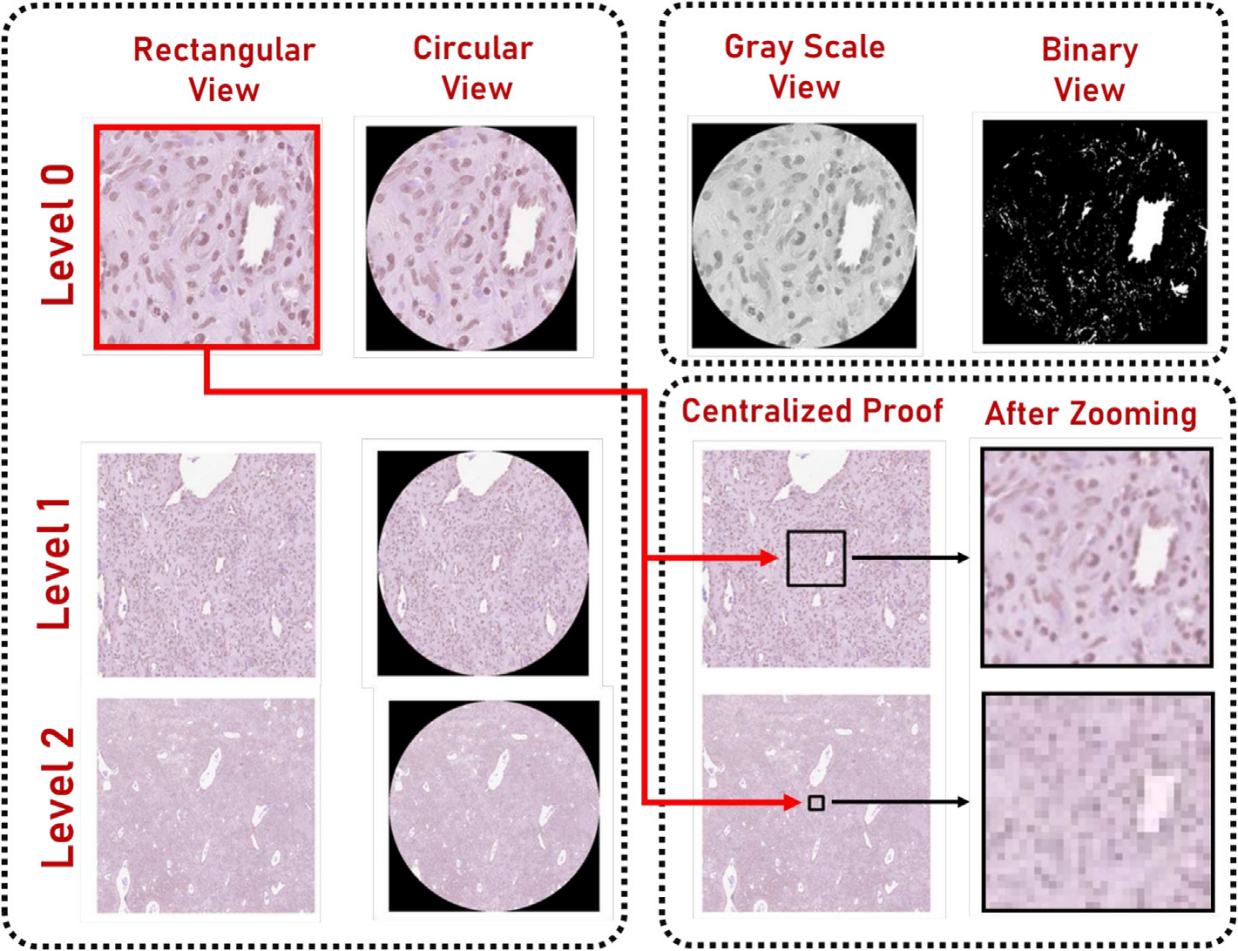
Graphical representation of the patch extraction and preprocessing, showcasing rectangular and circular patch views, grayscale and binary representations, and centralized batching alignment.

Aligned patches were extracted from three hierarchical levels to serve as the primary data for model training. Each patch underwent standardization to attain a dimension of (512 × 512) pixels. To enhance the dataset's quality, patches lacking discernible histological content or deemed to represent background noise were identified and filtered out. This was done by performing a thorough analysis of gray-level and binary representations of patches. This ensures only those patches which contain pathological information of interest are retained for further analysis. Patches at Level 0 were aligned to the midpoint point of Level 1, and Level 1 patches were aligned to the midpoint point of Level 2. This ensures that the patches at each level capture important spatial information. These processes are visually shown in Fig. 4. To ensure that all levels are centralized, we displayed the “After Zooming” subplots. This ensured that the three subplots should look alike but at different resolutions.

### ViT models optimization

The ViT model is an innovative neural network architecture with proven effectiveness in various image classification tasks.^15^^,^^16^ The major component of ViT is the multiple layers of self-attention modules, each followed by a feedforward neural network.^17^ Such an architecture enables the ViT to derive global contextual information from an input image, thus effectively understanding relationships between different regions.^18^^,^^19^ Several studies highlighted the applicability of the ViT model to specific aspects of histopathological image analysis, e.g., in brain imaging,^20^ breast pathology,^21^, and a large number of other domains.^22^, ^23^, ^24^

In this study, we have used the pre-trained ViT L/32 architecture model as our basic building block to further fine-tune. The major motivation for this selection was that the ViT model proved to be adequate to handle image data and particularly has proven efficiency in dealing with various spatial characteristics present in the histopathological image.^25^ We address variations in the significance of features across different resolutions by training three individual ViT models, each specialized in processing patches from a specific level, i.e., Level 0, Level 1, and Level 2. By tailoring these models to their respective spatial scales, we aimed to have the best performance of the models capturing the relevant features.^26^

Algorithm 1 indicates the basic steps of the ViT Model. As for the classification, we adopted the WMV scheme, which allowed us to obtain the final classification for each input image.^27^ We provided the integrated prediction from the three fine-tuned ViT models and assigned differential weights based on the confidence levels of these models. Based on such a complex interplay between fine-tuning ViT models and a weighted voting scheme, we could obtain a reliable and accurate classification result.^28^

Algorithm 1 Overview of the Key Steps of the ViT Process.

1: Divide the image into a sequence of 2D patches and then flatten.

2: Linear projections of flattened patches.

3: Addition of a learnable classification token and positional embedding matrix to the projected patches.

4: Feed both the patch and positional embedding matrix into the transformer encoder.

5: Pass the encoder output of the transformer through an MLP head and derive the ultimate prediction output.

## Experimental results

### Hardware configurations

The experiments were conducted on a high-performance computing platform equipped with a 12th Gen Intel(R) Core (TM) i9-12900 processor and 64 GB of memory. This system ran on a 64-bit Windows 11 Pro operating system, with graphics processing handled by an NVIDIA RTX A2000 12GB graphics card.

### Implementation details

The ViT model utilized in this study underwent hyperparameter tuning, achieving optimal parameters through training for 32 epochs with a learning rate of 0.001, utilizing the Adam optimizer. A patch size of 512 × 512 was consistently applied across all levels of the WSIs Pyramid. To ensure a robust evaluation of the model's performance and validate its reliability, two experiments were conducted: a train-test split and a 4-fold cross-validation (CV). Table 1 provides a detailed breakdown of the image and patch distribution for the MEN and SFT classes across both experiments. It also summarizes the total number of images and patches used in the study. All coding tasks were conducted using the Python programming language, utilizing the OpenSlide, and torchvision libraries.^29^^,^^30^

**Table 1:** Distribution of images and patches for MEN and SFT classes across train-test and 4-fold cross-validation experiments.

Experiment	Class	Number of images			Number of patches		
		Training	Validation	Testing	Training	Validation	Testing
Train-test	MEN	40	3	3	465,394	41,233	40,827
	SFT	40	3	3	396,013	37,591	32,797
4-fold cross-validation	MEN	36 (9 per fold)		10	440,725		106,729
	SFT	36 (9 per fold)		10	373,314		93,087
Total		92			1,013,855		

### Performance evaluation

The performance of the proposed approach is evaluated using a set of metrics, including accuracy (ACC), sensitivity (SEN), specificity (SPC), precision (PRC), balanced accuracy (BAC), receiver operating characteristic (ROC), and F1 score. All of these metrics along with their definitions and equations are presented in Table 2. In this table, TP denotes true positives, TN denotes true negatives, FP denotes false positives, FN denotes false negatives, and *N* represents the total number of instances.^31^ The evaluation process is based on patch-level predictions, which directly contribute to more reliable slide-level classification.

**Table 2:** Quantitative metrics and their definitions, along with corresponding equations utilized for performance evaluation.

Performance metric	Definition	Equation
Accuracy	The ratio of correctly predicted instances to the total instances in the dataset.	$\frac{\mathrm{TP}+\mathrm{TN}}{N}$
Sensitivity	The ratio of true positives to the sum of true positives and false negatives.	$\frac{\mathrm{TP}}{\mathrm{TP}+\mathrm{TN}}$
Specificity	The ratio of true negatives to the sum of true negatives and false positives.	$\frac{\mathrm{TN}}{\mathrm{TN}+\mathrm{FP}}$
Precision	The ratio of true positives to the sum of true positives and false positives.	$\frac{\mathrm{TP}}{\mathrm{TP}+\mathrm{FP}}$
Balanced accuracy	The arithmetic mean of sensitivity and specificity.	$\frac{1}{2}$ × (Sensitivity + Specificity)
ROC approximated	The average of sensitivity and precision.	$\frac{1}{2}$ × (Sensitivity + Precision)
F1-score	The harmonic mean of precision and sensitivity.	$2\times \frac{\text{Precision}\times \text{Sensitivity}}{\text{Precision}+\text{Sensitivity}}$

The proposed approach starts with patch-level classification, where each patch is classified based on its specified magnification level. Once we get the individual patch-level predictions, we combine them using a WMV scheme. The aim of this combination is to enhance the reliability of the patch-level predictions by incorporating complementary information from multiple levels, ensuring that each patch's final decision is robust. Eq. (1) mathematically expresses the WMV schema, where *C* represents the predicted class, *N* is the number of models, *w*_*i*_ is the weighted factor derived from the normalized accuracy of model *i*, and $P_{i}\left(C=c|X\right)$ is the probability assigned to class *c* by model *i* for input *X.*^32^

(1) $$\mathrm{WMV}=\arg\max_{c}\sum_{i=1}^{N}w_{i}\times P_{i}\left(C=c|X\right).$$

The weight factors $w_{i}$ are computed by evaluating each model on the entire dataset and then normalizing their accuracy. The normalized values are then used as weight factors for each class. The primary steps of the WMV scheme are outlined in Algorithm 2.

In the current study, each level was assigned a weight depending on its relative significance and performance in the classification task. Specifically, after experimenting with various weights using a grid search approach, we allocated weights of [0.365, 0.359, 0.276] to levels 0, 1, and 2, respectively. This process involved testing all possible combinations of weights for the three levels using all extracted patches, including a dedicated test set that remained entirely unseen during the training and validation phases. In more detail, two nested loops were constructed, where weights $w_{0}$ and $w_{1}$ iterated from 0 to 1 in increments of 0.001. The third weight, $w_{2}$ was computed as $w_{2}=1-\left(w_{0}+w_{1}\right)$, ensuring that the sum of weights remained equal to 1. To maintain this constraint, combinations where $w_{0}+w_{1}>1$ were skipped. This procedure allows for exhaustively searching all possible weight combinations, ensuring that more accurate models have a greater influence on the final patch decision, leading to a robust aggregation and a more reliable final decision for each patch.

**Figure:**
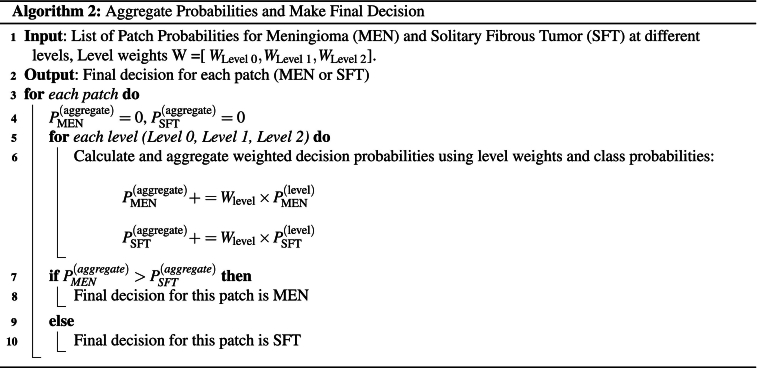

Results obtained from the proposed approach are outlined in Table 3, showing the performance of the ViT model at different levels of the WSI pyramid: Level 0, Level 1, and Level 2. In order to analyze the robustness and general applicability of the model, two separate validation methods were applied: train-test and 4-fold CV. This experimental design makes it possible to find the most robust level of image resolution and shows how the combination of different information from all levels can enhance the overall performance.

**Table 3:** Comparison of results for ViT model across different levels of WSI pyramid and the combined majority of all levels for train-test and 4-fold cross-validation.

Experiment	Levels	Evaluation metrics						
		Acc	SEN	SPC	PRC	BAC	ROC	F1-score
Train-test	Level 0	90.64%	93.97%	86.74%	89.27%	90.35%	91.62%	91.56%
	Level 1	89.26%	86.01%	93.07%	93.58%	89.54%	89.79%	89.63%
	Level 2	68.74%	42.36%	99.70%	99.41%	71.03%	70.88%	59.40%
	**Combined (Majority)**	**92.27%**	**87.77%**	**97.55%**	**97.68%**	**92.66%**	**92.73%**	**92.46%**
4-fold CV	Level 0	89.20%	88.76%	89.70%	90.81%	89.23%	89.78%	89.77%
	Level 1	89.67%	89.73%	89.61%	90.82%	89.67%	90.28%	90.27%
	Level 2	85.35%	92.82%	76.78%	82.09%	84.80%	87.45%	87.13%
	**Combined (Majority)**	**94.68%**	**96.05%**	**93.11%**	**94.11%**	**94.58%**	**95.08%**	**95.07%**

For the results of train-test experiment, Level 0 is the best among the other levels, with an accuracy of 90.64%, high sensitivity (93.97%), and a specificity of 86.74%. Level 1 is slightly lower than Level 0, but it also presents an accuracy of 89.26% with a good balance between sensitivity (86.01%) and specificity (93.07%). In contrast, Level 2 shows a drop in performance with an accuracy of 68.74% and a sensitivity of 42.36%, whereas performing very well in terms of specificity (99.70%). The combined majority of all levels improve the overall performance, reaching an accuracy of 92.27% and specificity of 97.55% with a balanced performance across all metrics.

For the results of 4-fold CV, Level 0 achieved a stable performance with 89.20% accuracy and a balanced sensitivity (88.76%) and specificity (89.70%). Level 1 has slightly higher results with 89.67% accuracy, high sensitivity of 89.73% and specificity of 89.61%. As in the case of the train-test results, Level 2 shows the accuracy with 85.35%, but still retains high sensitivity at 92.82% and performs well in the ROC and F1-score metrics. However, the combined approach raises the model performance, reaching an accuracy of 94.68% with substantial improvements in sensitivity at 96.05% and F1-score at 95.07%. These results underline the benefit of integrating information from multiple resolution levels.

**Fig. 5:**
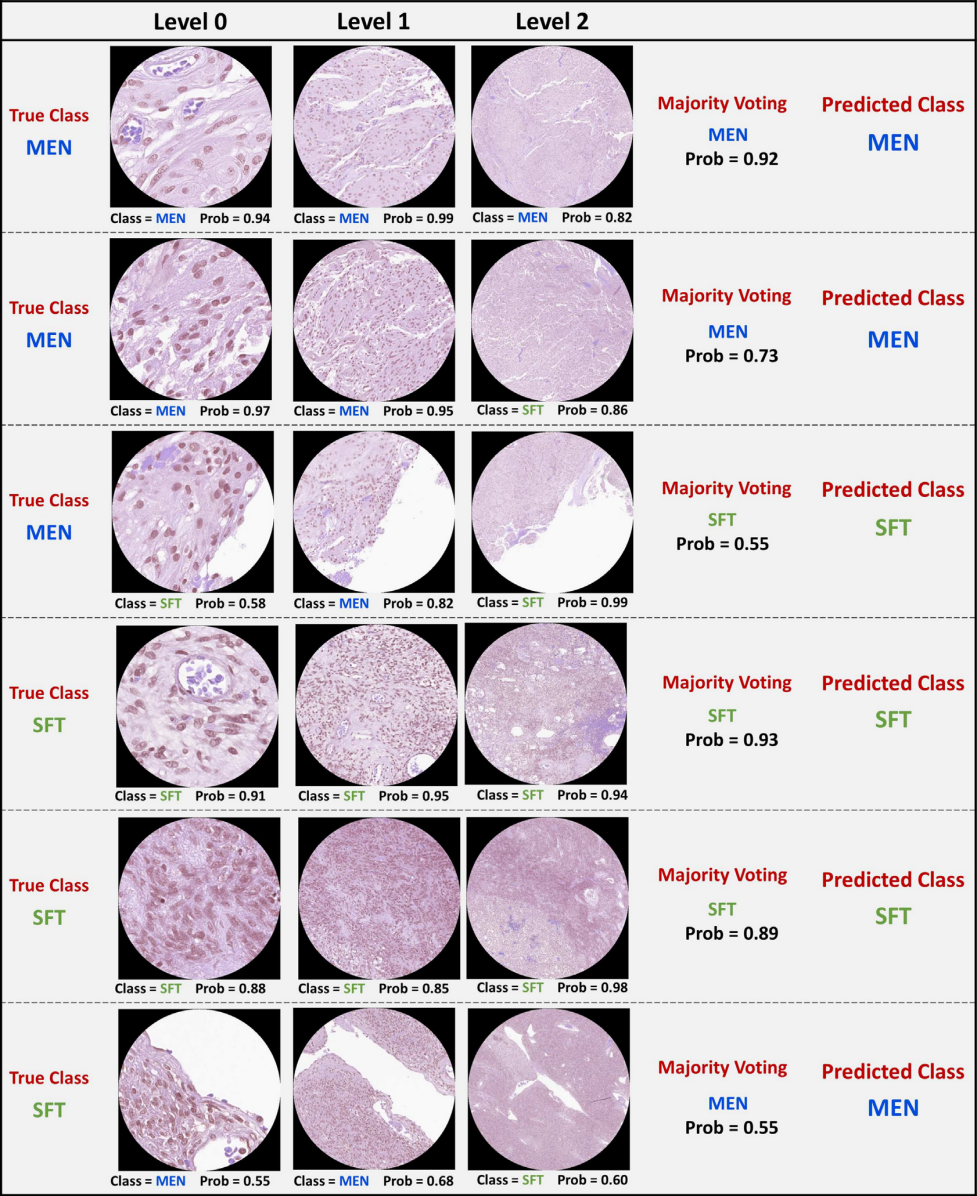
A graphical visualization of the model predictions across a set of patches at various levels within the WSI Pyramid. Each patch is labeled with its corresponding predicted class. Notably, the weights used for each level were [0.365, 0.359, 0.276], used in the calculation of the majority voting score that determined the final class decision.

An example of a graphical representation of model predictions is shown in Fig. 5, which illustrates the prediction of a set of patches at different pyramid levels of the WSI. It also shows the majority score, used to determine the final class decision, thus giving a clearer overview of how the model combines information from different resolution levels to make its predictions.

### Visualization of WSIs classification results

**Fig. 6:**
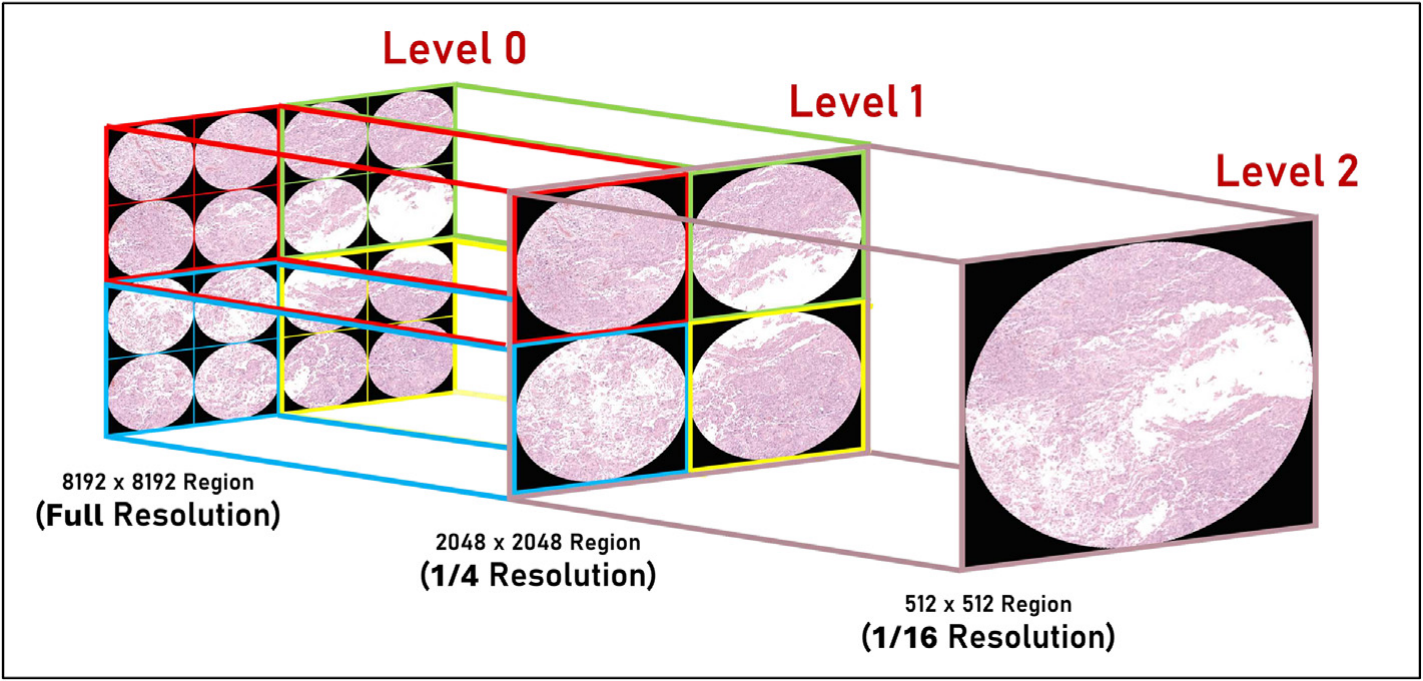
Visualization of the process of utilizing different WSI levels to get the final regional class decision.

To visually represent the classification outcomes applied to WSIs, we implemented the following patch classification procedure. This process involved aligning regions representing the same physical area but at various sizes and resolutions across multiple levels. Specifically, we aligned a region of size (8192 × 8192) at Level 0 with a region of size (2048 × 2048) at Level 1, and further aligned this with a region of size (256 × 256) at Level 2. Despite the differences in size, all regions provided an equivalent view due to their respective resolutions. Fig. 6 offers a visual representation of this process.

At each level, decisions were made by dividing the region of interest into patches of size (256 × 256), calculating decision probabilities for each patch, and then aggregating these probabilities to derive normalized probabilities representing the overall decision probabilities for each class. After collecting decisions from the three distinct levels, we aggregated the results by assigning weights to each level based on its classification performance. This led to the ultimate regional class decision. Subsequently, we applied this decisive classification to color the corresponding region giving precedence to the decision with the highest probability. This predefined procedure was uniformly applied to all WSI regions resulting in a complete coloring of the entire WSI.

### WSI prediction and explainability

For each WSI, a confidence score was computed as the average percentage of correctly classified patches over all regions and levels. In particular, for each region, the confidence score was obtained by taking the ratio between correctly classified patches over the total number of patches in that region. The WSI score was then obtained by averaging these regional scores over all the regions composing the WSI. This measure shows the model's consistency in aligning its predictions with the ground truth across different spatial areas at different resolution levels. The calculated confidence score was shown in the visualization for the quantitative assessment of the model's decisions on the whole WSI. Table 4 displays four WSIs. Two of them are for “MEN,” and the other two are for “SFT.” The WSIs go through the suggested system, which generates various outputs. These outputs include “Level 0,” “Level 1,” “Level 2,” “Combined,” and the original WSI. Blue represents MEN, and Green represents SFT. In the top left WSI, the system predicts a MEN diagnosis with 95% confidence. Moving to the top right WSI, it also predicts MEN but with a slightly lower confidence of 92%. Notably, the presence of Level 2 obstructed the system's ability to increase the confidence in this case. In the bottom left WSI, the system confidently diagnoses SFT with a confidence level of 100%. Finally, in the bottom right WSI, the system predicts SFT, but the confidence is lower at 51%, and it is worth mentioning that Level 0 had an inhibitory effect on raising the confidence in this instance.

**Table 4:**
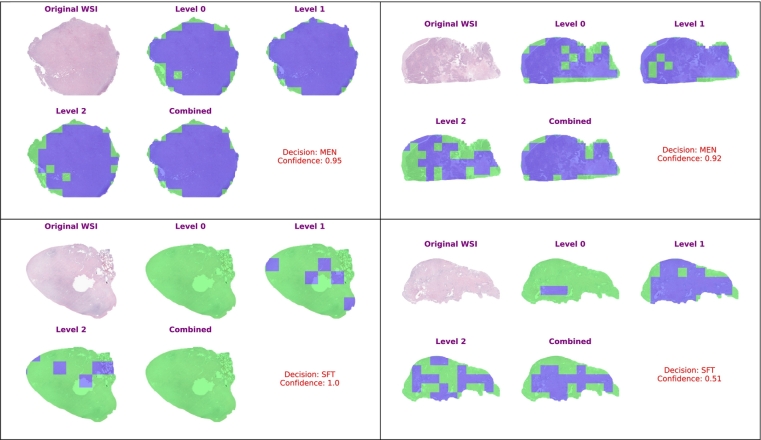
Visual presentation of four WSIs including “Level 0,” “Level 1,” “Level 2,” “Combined,” and the original WSI. Blue represents MEN, and Green represents SFT.

## Overall discussion

The present study addresses the challenging diagnostic task of distinguishing between MEN and SFT, two tumor types known for their overlapping morphological features. Conventional IHC-based approaches, while widely used, encounter limitations in resource-constrained settings and in scenarios involving frozen section analyses. In response to these challenges, we propose an approach leveraging the ViT architecture, in conjunction with multi-resolution processing, to improve histopathological analysis.

The model was initiated by extracting patches from various tiers within the WSI hierarchy for multi-resolution analysis. Extracted patches are processed in a circular manner to account for potential rotational variability. Patches containing restricted histological data were excluded retaining only high-quality patches for analysis. Patches at each level are fed into a separate Large/32 ViT model, and then the classification results from individual transformers are amalgamated using a WMV schema to determine the final predicted class. This approach ensures complete spatial coverage by exploiting different levels of the WSI hierarchy. The multi-scale analysis allows for subtler insight into the details at the highest resolution level and also incorporates wider contextual information, making the model capable of being evaluated from a more holistic perspective.

The results in Table 3 highlight the following observations: Level 0 always produces the best performance, with an accuracy of 90.64% in train-test and 89.20% in 4-fold CV. This shows that high-resolution patches are important for capturing small details that are relevant for good classification. Level 1 gives a good performance, too, with accuracy values of 89.26% and 89.67%, presenting a balanced sensitivity and specificity that indicates mid-resolution patches complement high-resolution information. Level 2 has lower accuracy, at 68.74% and 85.35%, but achieved specificity: 99.70% and 76.78%. Using only Levels 0 and 1 resulted in a slight performance decrease, regarding 2%–3% drop in accuracy and 3%–5% in sensitivity compared to the combined approach. This clearly shows the importance of Level 2 in adding a global view of the tissue, which can help in identifying larger structures and distinguishing non-tumor areas.

A notable improvement is observed when combining information from all WSI pyramid levels. The integrated approach leads to substantial performance enhancement across both evaluation methods. An accuracy of 92.27% and a specificity of 97.55% were achieved in the train-test experiment, whereas the 4-fold CV experiment shows further improvement, with an accuracy of 94.68%, along with a remarkable sensitivity of 96.05% and F1-score of 95.07%. These results confirm the synergy effect of multi-resolution data: The integration of information across all levels provides a more complementary understanding of the tissue, further boosting diagnostic performance. The stability and consistency of model performance in both evaluation methods is an indication of the robustness of the model, showing that it generalizes well and is consistent in different dataset partitions.

**Fig. 7:**
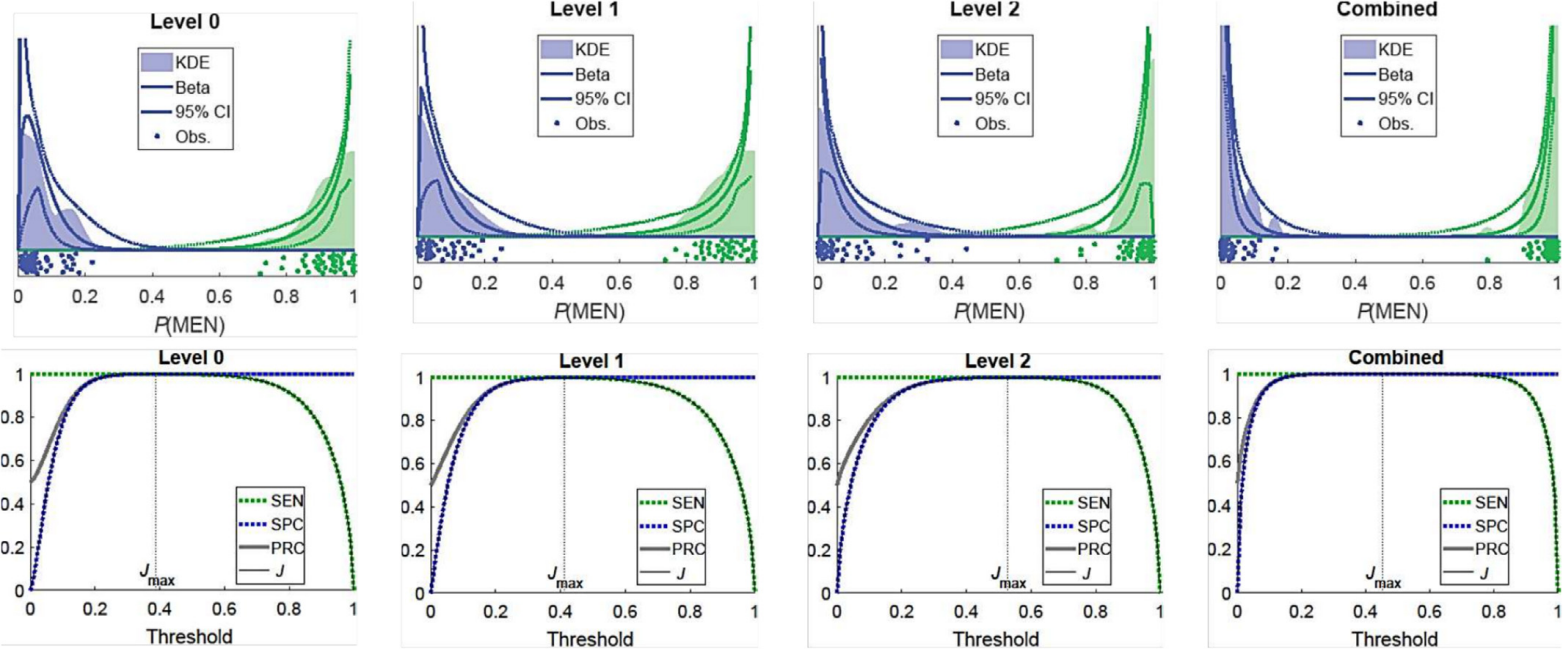
Probability distributions and performance metrics across individual levels and the combined decision approach. The top row depicts modeled probability density functions for *p*(MEN) for MEN (Blue) and SFT (Green) classes at Levels 0, 1, and 2 and combined. Also shown are 95% pointwise confidence bands on the densities (dotted lines) as well as kernel density estimates (shaded) for comparison. The bottom row shows corresponding estimates of sensitivity (SEN), specificity (SPC), precision (PRC), and Youden index (*J*) using these for WSI classification. Optimal decision threshold is labeled *J*_max_. (For interpretation of the references to color in this figure legend, the reader is referred to the web version of this article.)

To further assess the performance of our approach, The fraction of patches recognized as class MEN, *p*(MEN), was modeled as a beta distribution with shape parameters dependent upon the ground-truth diagnosis and zoom level. Values for the parameters were obtained as maximum-likelihood estimates given the output from the 4-fold CV experiment. From the model distributions we derive the theoretical SEN, SPC, PRC, and Youden index *J* for various thresholds of *p*(MEN) to classify the WSI. The results are illustrated in Fig. 7.

Bootstrapping was used to estimate 95% confidence intervals on the shape parameters. Each bootstrap sample was generated by selecting a random slide, with replacement, and setting *p*(MEN) to a random value drawn from a beta distribution with shape parameters $(n_{\mathrm{MEN}}+0.5,n_{\mathrm{SFT}}+0.5$), where *n*_*x*_ is the number of patches recognized as class *x* in the selected slide. One thousand bootstrap samples were used to estimate the 95% basic bootstrap confidence limits on each parameter. Performance metrics (SEN, SPC, and PRC) were modeled as Bernoulli probabilities for the purpose of estimating their confidence intervals, with all metrics exceeding 0.9545 with 95% confidence.

Additionally, another resampling strategy was used to evaluate the statistical significance of the aggregate decision being more accurate than the decision based on a single measurement scale, taken to be Level 0. For each bootstrap sample, a slide was selected at random from each ground-truth class. From these slides, tuples of single patch decisions (all scales plus the weighted aggregate) were resampled with replacement from the observed data. The false-positive rate for the SFT slide and the false-negative rate for the MEN slide were noted for each bootstrap sample and for both the Level 0 and aggregate decisions. The weighted aggregate is superior to the single scale decision with a lower rate of false positives (*p*=0.001) and false negatives (*p*=0.018). These findings underscore the statistical superiority of the weighted aggregate decision confirming that combining information from multiple levels is not only statistically significant but also clinically meaningful for reducing diagnostic errors.

## Limitations

Whereas promising results have been obtained with the proposed approach, some inherent constraints exist. Firstly, although there was an effort to build a multi-institutional dataset, the number of cases is limited and does not encompass the morphological heterogeneity of MENs and SFTs. Therefore, enlarging the dataset to include rare morphological subtypes and adequate representation of tumors of all grades is essential to determine the reliability of the generalization of the results. Another issue is the presence of other rare tumors in CNS locations that are neither MENs nor SFTs, such as sarcomas and sarcomatoid carcinomas, which can be part of the differential diagnosis. If such a case is presented to the classifier, it will provide an answer of either SFT or MEN, which is a limitation that needs to be clearly communicated. A more appropriate classifier for clinical translation would include categories for MEN, SFT, or neither. Additionally, it is important to note that although the tissues representing tumors in WSIs typically constitute 80%–90% of the image content, the model was trained on unannotated data, and non-diagnostic or irrelevant tissues were present among examples of MENs and SFTs in the datasets. Incorporating annotated data could enhance the accuracy and reliability of the model.

## Conclusions and future directions

The conclusion of this work underlines the significant benefits of the proposed approach in improving the discriminative ability to distinguish between MEN and SFTs. In combination with the multi-resolution processing strategy, the ViT architecture provides a robust solution to this diagnostic task. The proposed model processes different tiers of the WSI hierarchy showing good performance at various levels of abstraction, hence demonstrating the high potential of transformer models to provide significant improvements in the analysis of histopathological images. Such aggregation of decisions through a WMV scheme with all the strengths of each level compensates for their respective limitations, achieving an overall high accuracy of 94.68%, thus mitigating potential biases and significantly improving the reliability of the diagnoses. The proposed method provides a promising supplementary approach to conventional IHC-based methods, especially in resource-constrained environments and in time-sensitive analyses. Thus, such results reveal the significant potential of transformer models in providing highly accurate and reliable diagnostic support.

Future directions include extending the database to cover a more general variety of tumors and diverse patient demographics. Further, collaboration with clinical experts and pathologists, validating the suggested methodology for the collaboration and fine-tuning work, will inevitably provide smooth integration into the daily clinical routine. Finally, exploration of the applicability of explainable AI methods, which may provide an interpretable vision of the decision-making process of the proposed model, is of paramount importance for securing trust and confidence in the diagnosing outcomes.

## Declaration of competing interest

The authors declare that they have no known competing financial interests or personal relationships that could have appeared to influence the work reported in this article.
